# Evaluation of the Oral Health Conditions and Oral Health-Related Quality of Life in a Community-Dwellers Population Aged ≥ 45 Years in the Canton of Bern: A Preliminary Pilot Study

**DOI:** 10.3390/ijerph20054557

**Published:** 2023-03-04

**Authors:** Andrea Roccuzzo, Roberta Borg-Bartolo, Martin Schimmel, Christian Tennert, David J. Manton, Guglielmo Campus

**Affiliations:** 1 Department of Restorative, Pediatric and Preventive Dentistry, School of Dental Medicine, University of Bern, 3010 Bern, Switzerland; 2 Department of Periodontology, School of Dental Medicine, University of Bern, 3010 Bern, Switzerland; 3 Department of Reconstructive Dentistry and Gerodontology, School of Dental Medicine, University of Bern, 3010 Bern, Switzerland; 4 Division of Gerodontology and Removable Prosthodontics, University Clinics of Dental Medicine, University of Geneva, 1205 Geneva, Switzerland; 5 Centre for Dentistry and Oral Health, University Medical Centre Groningen, 9713 AV Groningen, The Netherlands; 6 Department of Surgery, Microsurgery and Medicine Sciences, School of Dentistry, University of Sassari, 07100 Sassari, Italy

**Keywords:** oral health, epidemiology, clinical trial, tooth loss

## Abstract

Objective: To evaluate oral health conditions and oral health-related quality of life in community-dwellers aged ≥ 45 years in the Canton of Bern, Switzerland. Materials and Methods: One hundred subjects (63% males; mean age: 73 years), selected randomly using a cluster procedure within the Canton of Bern, underwent a clinical oral examination after completing questionnaires on socio-economic level, medical history, oral health behaviour, and Geriatric Oral Health Assessment Index (GOHAI). Descriptive analyses and multinomial regression models were applied to investigate the association between oral health diseases (dental caries and periodontitis) and specific participant characteristics. Results: The mean number of decayed, missing, and filled teeth (DMFT) was 0.30, 4.20, and 8.75, respectively; the mean DMFT score = 13.35. Dental caries prevalence (ICDAS > 0) was 15% and periodontitis prevalence was 46%. Logistic regression models revealed that living in an urban area was associated with lower odds (OR 0.03, _95%_CI 0.00–0.36) of having periodontal disease. Male gender was associated with lower odds for dental caries (OR 0.31, _95%_CI 0.09–1.01) and total lack of professional tooth cleaning was associated with higher odds for dental caries (OR 41.99, _95%_CI 0.01–0.38). Ordinal logistic regression revealed that both the presence of dental caries (RR 12.80, _95%_CI 1.47–111.20) and periodontal disease (RR 6.91, _95%_CI 1.16–84.00) were statistically significantly associated with rheumatoid arthritis. Conclusion: Within the study limitations, untreated dental caries and periodontal disease are prevalent in the Swiss population, despite the high level of self-performed oral hygiene and access to the dental care system.

## 1. Introduction

It is widely accepted that oral health conditions are related to general health and that dental status impacts overall quality of life [1]. Due to the increasing number of elderly people as a consequence of demographic changes occurring worldwide [2], the identification of oral health conditions and the factors that might have an impact on oral health is highly relevant [3].

During the last three decades, even-tough trends for the improvement of the overall number of functioning dental units and periodontal conditions have been documented [4], dental caries and periodontal disease still represent the two main causes of tooth loss; consequently, early diagnosis and appropriate treatment is of paramount importance [5]. In Switzerland, as in most industrialized countries, the overall number of missing teeth and, more specifically, the number of totally edentulous people, has decreased [6] despite increasing life expectancy [7]. Ideally, full-mouth oral examination surveys should be performed to determine oral health conditions within populations, however, these analyses are not always feasible since they are very expensive and time-consuming [8].

When focusing on oral-health epidemiological data collected within the Swiss population during the last three decades, besides self-reported data [9] and two studies that assessed the periodontal condition in the elderly [4,10], no further evidence has been gathered with respect to oral health conditions, dental caries, and patient satisfaction.

Due to the paucity of data regarding oral health in Switzerland, the aim of the present study was to investigate dental caries experience and periodontal disease characteristics using clinical oral examinations and self-reported questionnaires in a representative randomly selected cohort of individuals within the Canton of Bern, Switzerland.

## 2. Materials and Methods

The study protocol was approved by the Ethical Committee of the Canton of Bern (KEK), Switzerland (Nr. 2020-02760). The investigation was conducted according to the revised principles of the Helsinki Declaration (2013), and signed informed consent was obtained from each participant before entering the study. Data reporting was according to the STROBE guidelines.

### 2.1. Study Design and Population

This is a cross-sectional, monocentric study within the general population aged ≥ 45 years of the Canton of Bern, Switzerland. Participants were randomly selected using a cluster procedure considering each village/town of the Canton as a cluster [11].

Sample size calculation was performed assuming an overall prevalence of oral diseases (dental caries and periodontitis) of 50% [12], a standard error of 0.05, and a design effect of 2.5 plus an increase of 10%, according to a methodology previously validated [13].

Consequently, the estimates were safeguarded at an optimal level of precision (5%) against the possible effect of (a) oral condition prevalence modification and (b) the number of non-responders; and the theoretical sample size was 728 subjects. For the present pilot investigation, a *post-hoc* power analysis, performed assuming a confidence of 0.95, and a probability of 0.5, led to 89 subjects [14]. To compensate for non-responders, the number of subjects was increased to 100.

### 2.2. Eligibility Criteria

Inclusion—Participants aged at least 45 years-of-age, living in the community, who had the capacity to understand the questionnaire items and provide a written informed consent form. Exclusion—Persons with known allergic reactions to oral hygiene products and/or medication and/or dental materials previously used in the mouth or pharynx, or who had pathological changes of the oral mucosa, e.g., acute ulcerating gingivitis, acute herpetic gingivostomatitis, recurrent aphthous ulceration, or systemic illnesses with oral manifestations.

### 2.3. Patients’ Recruitment, Screening, and Informed Consent Procedure

Proportional allocation was carried out to sample the individuals from the ten different regions of the canton of Bern, which were selected by probability proportional to size sampling, according to the proposed STEP approach guidelines [15]. If a town or village refused to participate, the next town/village was contacted, with participants being selected randomly. Before being enrolled, each participant gave written consent to participate in the study. The personal contact information of the citizens was provided by the municipalities which had agreed to participate in the study. The participants were contacted by mail and a printed information sheet with study details (objectives, benefits, potential risks, participants’ rights, and obligations) was provided. To those who agreed to participate in the study, detailed study information, questionnaires, and informed consent forms were mailed. They were also given an appointment for the clinical oral examination, which would take place at the participant’s home.

The informed consent form and the declaration of data protection comprised an integral part of the study documentation and are stored in dedicated secure archives by the principal investigator for a period of at least 15 years. No compensation or payment was planned or given to study participants.

### 2.4. Study Data Collection and Clinical Assessment

Prior to the clinical oral examination, participants completed the validated questionnaires on socio-economic status, medical history, oral health behaviours [16], and the Geriatric Oral Health Assessment Index (GOHAI) [17]. All questionnaires were made available in German, French, and Italian. All clinical examinations were performed by four senior-year dental students under the strict supervision of two experienced clinicians (AR—experienced board-certified periodontist and RBB—restorative dentist) using a plain mirror (Hahnenkratt, Knigsbach, Germany) and WHO ball-ended probe (Asa-Dental, Milan, Italy) under artificial light.

Prior to the initiation of the study, a clinical calibration on participants was not possible due to the COVID-19 outbreak, therefore, a two-session calibration was performed using twenty clinical photographs, with each image showing different stages of dental caries and/or different dental restorations. With respect to the assessment of the periodontal conditions, where calibration was not possible using clinical photographs, the examiners were individually trained. Kappa scores were calculated for intra-rater reliability while intra-class correlation coefficients (ICC) using the two-way mixed effects model were calculated for inter-rater reliability, against the benchmark examiner (RBB). Kappa scores and ICC were calculated for both dental caries and dental restorations, with ICC being calculated for both calibration sessions.

The following clinical indices were collected:

▪ ICDAS Codes: caries lesion data was recorded using the two-digit codes related to ICDAS for each tooth surface [18].
▪ Simplified Papilla Bleeding index (PBI): all mesial and distal interproximal spaces were examined using a periodontal probe and recorded as positive if bleeding was detected after gentle probing (0/1) [19].
▪ Plaque Index inter-proximal Space (Approximal Plaque Index): all mesial and distal interproximal spaces were examined and recorded as positive if plaque was detected visually (0/1) [20].
▪ Periodontal Screening Record (Periodontal Screening Index—PSI): six sites per tooth present per sextant were assessed and the highest score for each sextant was recorded. The scores range from 0 (healthy periodontal tissue) to 4 (probing pocket depth > 5 mm) [21].
▪ Presence of prosthetic rehabilitation (0/1) and assessment of the type of reconstruction (partial fixed dental prostheses; implant-supported partial/total fixed dental prostheses; partial/total removable prostheses).
▪ Number of occlusal functional units (OFUs). One OFU was defined as a pair of opposing natural or prosthetic teeth, excluding third molars (0–14) [22].

Study data were anonymously stored and managed using dedicated software (REDCap^®^, Vanderbilt University, USA). Participants were grouped into three age categories: 45–64 years, 65–74 years, and ≥75 years. Socio-economic status was assessed using civil status, education, and employment levels. Location was divided into rural and urban with the cut-off taken to be at least 10,000 inhabitants for an area to be considered urban [6]. Data on the smoking status and alcohol status (0/1) was collected with the most commonly reported medical conditions, namely, high blood pressure, having or a history of cancer, cardiovascular disease, rheumatoid arthritis, depression, and Type 2 diabetes. Oral hygiene habits were assessed by recording the daily patient-reported tooth brushing frequency, type of toothbrush, use of fluoridated toothpaste, mouthwash and interdental brushes/dental floss, and frequency of visits to the dentist and dental hygienist. The Geriatric Oral Health Assessment Index (GOHAI) scores were categorized into two groups: GOHAI < 45 and GOHAI ≥ 45 (45 being the average GOHAI score obtained). Dental caries was divided into having initial carious lesions (ICDAS 1–3) and dentine carious lesions (ICDAS 4–6) [23], while periodontal disease was categorized as not having periodontal disease (PSI 0–2) and active periodontal disease (PSI 3–4).

### 2.5. Data Analysis

The intra-examiner reliability Kappa scores ranged from 80–100% (*p* < 0.05) for dental caries lesion calibration, whilst 100% (*p* < 0.05) intra-examiner reliability was achieved by all examiners for the dental restoration calibration. The average ICCs for inter-rater reliability were 0.97 (_95%_CI = 0.94–0.98, *p* < 0.05) (dental caries, first calibration session), 0.95 (_95%_CI = 0.90–9.98, *p* < 0.05) (dental caries, second calibration), and 0.98 (_95%_CI = 0.97–0.99, *p* < 0.05) (dental restoration, first and second calibration).

Bivariate analysis between clinical data and questionnaire items (age and socio-economic variables, the most reported medical conditions, oral health behaviours, oral health-related quality-of-life, and oral health conditions) was run. Chi-square tests or Fisher tests (if a count was less than five) were assessed. The presence of a trend between clinical data and questionnaire items was evaluated using the Cochrane–Armitage trend test for variables with a binary outcome and Cuzick’s test for trend for variables with an ordered outcome including Montecarlo permutations for a more precise outcome. Logistic regressions were carried out to analyse the relationship between i) the presence of periodontal disease ii) dental caries (dentine carious lesions), and age, socio-economic factors, medical conditions, oral hygiene, and oral health behaviours, and odds ratios were calculated. A multinomial ordinal logistic regression was also performed to analyse the relationship between being free of any oral diseases (i.e. no caries lesions (ICDAS < 4), no periodontal disease), having at least one disease (i.e. no caries lesions (ICDAS < 4), yes periodontal disease or yes caries lesions (ICDAS < 4), no periodontal disease) or having both diseases with age, socio-economic factors, medical conditions, oral hygiene, and oral health behaviour. Effect modifiers were identified via stratum-specific measures and the analysis was modified accordingly.

Statistical analysis was performed using Stata SE17^®^ (StataCorp LLC, College Station, TX, USA) with statistical significance set at *p* < 0.05.

## 3. Results

A total sample of 100 participants over the 1430 originally reached by mail (response rate = 7.00%) was included with a majority of males (n = 63) (*p* = 0.05) and a mean age of 73 years (SD = ±10.77) (range: 45–99). The majority lived in rural areas (n = 53), were married (n = 67), and retired (n = 66). No statistically significant differences among the groups were detected with respect to the region of residency, civil status, and educational level (*p* > 0.05). When focusing on the medical conditions, 37% of the individuals reported high blood pressure, while 68.80% (n = 11) of the group ≥ 75 years reported being affected by cardiovascular disease (*p* = 0.03). Most participants (n = 89) were non-smokers (*p* = 0.06).

Home care oral health procedures were performed by most participants (n = 64) twice per day with a similar distribution with respect to the method (n = 48 vs. 62; manual vs. electric toothbrush, respectively), use of fluoridated toothpaste by 85 subjects, also adjunctive interproximal cleaning devices were used by more than two-thirds of participants.

All participants had at least one dental visit in the previous two years with the majority (n = 65; 81.30%) reporting having visited the dentist less than one year apart (*p* = 0.05).

The mean number of decayed, missing, and filled teeth was 0.30 (SD = ±1.07, range 0–8), 4.20 (SD = ±4.93, range 0–28), 8.75 (SD = ±4.98, range 0–19), respectively. The mean DMFT score was 13.35 (SD = ±5.38, range 1–28). There was an increasing trend of having missing teeth (*p* = 0.03) as people aged and a decreasing trend in the number of filled teeth (*p* < 0.01). The overall mean number of root caries lesions was 0.20 (SD = ±1.02, range 0–8). Sixteen participants had carious teeth with ICDAS 1–3 lesions while nine participants presented with ICDAS 4–6 lesions. Six participants had root caries lesions classified as ICDAS 2. Overall, 15% of the participants had at least one carious tooth, with 53 participants having a DMFT score of ≤13. Among the 100 participants, 26 had a maximum of four filled teeth, while 39 had at least 11 filled teeth (*p* = 0.02). The assessment of periodontal conditions revealed the presence of periodontitis (i.e., PSI scores 3–4) in half of the subjects (46%) with no statistically significant difference between groups (*p* = 0.47).

Eleven participants, all aged more than 75 years, were wearing removable prostheses, while 81 participants had fixed prostheses (*p* = 0.02); implant-retained prostheses were present in 22% of subjects. Finally, when focusing on the GOHAI scores, no statistically significant differences between the three groups were detected (*p* > 0.05) (Table 1).

The results of the logistic regression model revealed that living in an urban area was associated with lower odds of having periodontal disease than living in a rural region (OR 0.03; CI: 0.00–0.36).

Male gender was protective for having dental caries (OR 0.31; CI 0.09–1.01) while the total lack of professional tooth cleaning was statistically significantly associated with an increased risk for untreated caries lesions (OR 41.99; CI 0.01–0.38) (Table 2).

The results of the multinomial ordinal logistic regression analysis revealed that living in an urban compared to a rural location was statistically significantly associated with a lower relative risk of having periodontal disease (RR 0.33, CI 0.12–0.92), whilst the presence of untreated dental caries lesions or periodontal disease were statistically significantly associated with an increased relative risk ratio of 12.80 (CI: 1.47–111.20) and 6.91 (CI 1.16–84.00), respectively, in participants with rheumatoid arthritis. The results of having both dental caries and periodontal disease were excluded since only four participants were categorized as such (Table 3).

## 4. Discussion

The present study was planned to report preliminary data on the oral health status of a representative cohort of participants aged ≥ 45 years living in the Canton of Bern, Switzerland. Despite the increasing body of evidence regarding worldwide oral health [24], still, only limited data are available from several industrialized countries such as Switzerland. Even though nowadays, as reported by the Swiss National Statistics Office, 60% of the Swiss population is aged 40–64 years-of-age and an additional 1.6 million (18.9%) > 65 years, the available epidemiological data are scarce and existing data predominantly has no associated clinical examination [6]. Therefore, despite the pilot nature of the present study, the presented data have a unique value.

When analysing the prevalence of untreated caries lesions, 15% of the subjects have been diagnosed as having been affected by such disease (ICDAS score 4–6). To the best of the authors’ knowledge, these are the first caries data collected within Switzerland and therefore, comparison with previous data is not possible - nevertheless, when comparing the prevalence with the data from adjacent countries such as Germany and France, the present data indicate slightly lower caries experience [25,26]. The advantage of using ICDAS for recording the extent of caries lesions over other commonly used methods, such as the DMFT index, is that with ICDAS the severity and prevalence of caries can be reported in its continuum [18] enabling better preventive and treatment programs, tailored to the needs of the population [27].

Periodontal disease still represents the second most common reason for tooth loss, after dental caries [28]. Historical longitudinal data from the same geographic area (Canton of Bern) from 2010, revealed that periodontal disease affected only 11% of the examined subjects and 2% of the examined sites [4], to the contrary, in the present study almost half of the participants were diagnosed with periodontal disease (PSI Score 3–4). One reason for this discrepancy is the diagnostic tool used to detect the disease-indeed, previously full-mouth periodontal parameters were recorded [4], whilst in the present study, the deepest periodontal probing depth at tooth-site per sextant was chosen as representative of the periodontal condition of that specific sextant. When focusing on plaque and bleeding scores, a generalised increasing trend when comparing the three age groups was noted. Indeed, it has been extensively reported that elderly patients have higher plaque accumulation and associated bleeding due to their difficulties in performing appropriate oral hygiene procedures [29]. Another interesting finding is that rheumatoid arthritis was found to be statistically significantly associated with having untreated caries lesions and periodontal disease in the present study. With respect to periodontal disease and rheumatoid arthritis, these results are in line with previous publications, which reported challenges in the maintenance of good oral hygiene [30] and suggested a relationship between these two chronic inflammatory conditions [31]. Furthermore, the present data are confirmatory of previous studies which pointed out the strong association between periodontitis, smoking, and diabetes [32].

When focusing on smoking habits, less than 10% of subjects reported being smokers, reflecting the high level of awareness amongst Swiss people aged ≥ 45 years-of-age of the harmful health effects of smoking. This proportion is considerably lower than those reported in the literature [33] where it is estimated that the prevalence of tobacco smokers is around 25%. A putative explanation is that the adults and elderly patients have quit smoking during life due to the progressive (risk of) onset of chronic diseases (i.e. cardiovascular diseases and hypertension). These results confirm a recent trend published by the Swiss National Statistics Office of around 50% of men and 30% of females aged ≥65 years being former smokers [34].

One further aspect that should be underlined is that greater than 80% of participants reported performing tooth-brushing at least twice-per-day and routinely using adjunctive devices (i.e. dental floss and/or interdental brushes). This information, combined with the frequent visits to the dentist and/or dental hygienist for professional tooth cleaning, indicates the high level of awareness of the importance of oral hygiene procedures and might provide indirect evidence of the quality of the oral health care system in Switzerland [9]. More specifically, the Swiss Social Care System covers the yearly cost of one session of professional tooth cleaning for those subjects who cannot afford it [35].

Historically, to restore missing teeth, partial fixed/removable dental prostheses have been used [36]. However, during the last three decades, the introduction of dental implants has changed the method for rehabilitating these patients radically [37,38]. The present study confirms this trend within Switzerland [6]; indeed, 22% of the participants had implant-supported restorations and only 11% were wearing “traditional” removable prostheses.

During the past two decades, patient-related outcome measures (PROMs) have gained popularity; in particular, specific validated questionnaires such as the GOHAI have been used to evaluate oral health conditions from the patients’ perspectives. In the present study, the mean GOHAI score was 45 which is lower than those reported in neighbouring countries [39,40].

The present study has some strengths and limitations: the complex randomization process to detect the target population and the presence of a clinical examination performed by two experienced and previously calibrated dentists should be acknowledged as strengths, whilst the limited number of participants, the low response rate, and questionnaire sourced data are limitations. Consequently, the data gathered from the present pilot study are not generalizable to the Swiss population.

## 5. Conclusions

Within the limitations of the present pilot study, despite the high awareness of the importance of home care plaque control and the reported frequent visits to a dentist, dental caries and periodontitis are still highly prevalent conditions in adults and elderly Swiss subjects.

## Figures and Tables

**Table 1:** Participant characteristics among the three different age groups.

Variable	45–64 yy	65–74 yy	>75 yy	Participants	Fisher’s Exact Test	Trend	
	*n* = 15	*n* = 39	*n* = 46	*n* = 100	*p*-Value	z Value	*p*-Value
**Sex**	*n* (%)	*n* (%)	*n* (%)	*n* (%)			
Female	11 (29.73)	14 (37.84)	12 (32.43)	37.00 (100)			
Male	4 (6.35)	25 (39.68)	34 (54.97)	63.00 (100)	**0.01**	3.03	**0.01**
**Location**							
Urban	7 (14.89)	22 (46.81)	18 (38.03)	47 (100)			
Rural	8 (15.09)	17 (32.08)	28 (52.83)	53 (100)	0.29	−0.10	0.38
**Civil status**							
Married	13 (19.40)	26 (38.81)	28 (41.79)	67 (100)			
Not married	2 (6.67)	13 (43.33)	15 (50.00)	30 (100)	0.29	−1.33	0.18
**Education level**							
Primary-secondary education	7 (22.58)	11 (35.48)	13 (41.94)	31 (100)			
Tertiary education	6 (12.24)	21 (42.86)	22 (44.90)	49 (100)			
No education/unknown	2 (10.00)	7 (35.00)	11 (55.00)	20 (100)	0.67	1.26	0.23
**Employment level**							
In employment	11 (78.57)	3 (21.43)	0 (0.00)	14 (100)			
Retired	1 (1.52)	30 (45.45)	35 (53.03)	66 (100)			
Unknown	3 (15.00)	6 (30.00)	11 (55.00)	20 (100)	**<0.01**	3.97	**<0.01**
**Smoking status**							
Non-smoker	15 (16.85)	37 (41.57)	37 (41.57)	89 (100)			
Smoker	0 (0)	2 (25.00)	6 (75.00)	8 (100)	0.27	−1.90	0.06
**Alcohol status**							
Do not drink alcohol	7 (14.58)	20 (41.67)	21 (43.75)	48 (100)			
Drink alcohol regularly	8 (15.38)	19 (36.54)	25 (48.08)	52 (100)	0.89	0.25	0.91
**Medical history**							
High blood pressure	4 (10.81)	14 (37.84)	19 (51.35)	37 (100)	0.62	1.02	0.39
Had/have cancer	0 (0)	9 (52.94)	8 (47.06)	17 (100)	0.12	1.01	0.41
Cardiovascular disease	0 (0)	5 (31.25)	11 (68.75)	16 (100)	0.07	2.29	**0.03**
Rheumatoid arthritis	0 (0)	5 (38.46)	8 (61.54)	13 (100)	0.24	1.65	0.14
Depression	0 (0)	6 (50.00)	6 (50.00)	12 (100)	0.31	0.98	0.46
Diabetes	0 (0)	6 (54.55)	5 (45.45)	11 (100)	0.35	0.71	0.65
**Tooth brushing frequency**							
Once daily	1 (5.88)	3 (17.65)	13 (76.47)	17 (100)			
Twice daily	12 (18.75)	25 (39.06)	27 (42.19)	64 (100)			
More than twice daily	2 (10.53)	11 (57.89)	6 (31.58)	19 (100)	0.05	−1.98	0.06
**Type of toothbrush**							
Manual	10 (20.83)	15 (31.25)	23 (47.92)	48 (100)			
Electric	5 (9.62)	24 (46.15)	23 (44.23)	52 (100)	0.17	0.53	0.71
**Use of fluoridated toothpaste**							
No	3 (23.08)	4 (30.77)	6 (46.15)	13 (100)			
Yes	12 (14.12)	33 (38.82)	40 (47.06)	85 (100)	0.66	0.46	0.79
**Use of mouthwash**							
No	5 (16.13)	11 (35.48)	15 (48.39)	31 (100)			
Yes	10 (14.48)	28 (40.58)	31 (45.93)	69 (100)	0.88	−0.12	1.00
**Use of dental floss/interdental brushes**							
No	2 (15.38)	4 (30.77)	7 (53.85)	13 (100)			
Yes	11 (16.18)	28 (41.18)	29 (42.65)	68 (100)	0.85	−0.55	0.75
**Last visit to the dentist**							
<1 year	10 (15.38)	27 (41.54)	28 (43.08)	65 (100)			
1–2 years	3 (23.08)	5 (38.46)	5 (38.46)	13 (100)			
>2 years	0 (0.00)	0 (0.00)	2 (100)	2 (100)			
Never	0 (0.00)	0 (0.00)	0 (0.00)	0 (100)	0.74	0.06	0.95
**Last visit to the dental hygienist**							
<1 year	10 (15.38)	29 (44.62)	26 (40.00)	65 (100)			
1–2 years	2 (22.22)	3 (33.33)	4 (44.44)6	9 (100)			
>2 years	1 (50.00)	0 (0.00)	1 (50.00)	2 (100)			
Never	0 (0.00)	0 (0.00)	4 (100)	4 (100)	0.21	0.90	0.37
**Approximal Plaque Index (API)**							
≤25%	11 (25.58)	20 (46.51)	12 (27.91)	43 (100)			
25%–75%	2 (4.88)	15 (36.59)	24 (58.54)	41 (100)			
>75%	0 (0.0)	3 (27.27)	8 (72.73)	11 (100)	**<0.01**	3.86	**<0.01**
**Papilla Bleeding index (PBI)**							
≤25%	11 (16.42)	29 (43.28)	27 (40.30)	67 (100)			
25%–75%	0 (0.00)	7 (33.33)	14 (66.67)	21 (100)			
>75%	1 (20.00)	2 (40.00)	2 (40.00)	5 (100)	0.08	1.91	0.05
**Periodontal disease**							
No periodontal disease	10 (19.23)	20 (38.46)	22 (42.31)	52 (100)			
Periodontal disease	5 (11.11)	19 (42.22)	22 (46.67)	4 (100)	0.52	0.85	0.47
**Dental caries**							
Initial carious lesions	14 (16.47)	31 (36.47)	40 (57.06)	85 (100)			
Dentine carious lesions	1 (6.67)	8 (53.33)	6 (40.00)	15 (100)	0.41	0.14	1.00
**Missing teeth**							
No missing teeth	4 (36.36)	3 (27.27)	4 (36.36)	11 (100)			
≤10 missing teeth	11 (13.75)	35 (43.75)	34 (42.50)	80 (100)			
>10 missing teeth	0 (0.00)	1 (11.11)	8 (88.89)	9 (100)	**0.03**	2.67	**0.01**
**Filled teeth**							
0–4 filled teeth	7 (26.92)	2 (7.69)	17 (65.38)	26 (100)			
5–10 filled teeth	4 (11.43)	17 (48.57)	14 (40.00)	35 (100)			
>10 filled teeth	4 (10.26)	20 (51.28)	15 (38.46)	39 (100)	**<0.01**	−0.51	0.62
**DMFT-index**							
DMFT ≤ 13	12 (22.64)	20 (37.74)	21 (39.62)	53 (100)			
DMFT > 13	3 (6.38)	19 (40.43)	25 (53.19)	47 (100)	0.07	2.08	**0.05**
**Presence of removable prosthesis**							
No	15 (16.85)	39 (43.82)	35 (39.33)	89 (100)			
Yes	0 (0.00)	0 (0.00)	11 (100.00)	11 (100)	**<0.01**	3.38	**<0.01**
**Presence of fixed prosthesis**							
No	8 (42.11)	4 (21.05)	7 (36.84)	19 (100)			
Yes	7 (8.64)	35 (43.21)	39 (48.15)	81 (100)	**<0.01**	2.45	**0.02**
**Implant-retained prosthesis**							
No	13 (16.67)	27 (34.62)	38 (48.72)	78 (100)			
Yes	2 (9.09)	12 (54.55)	8 (36.36)	22 (100)	0.28	−0.28	0.91
**Occlusal functional Units (OFUs)**							
0–5 OFUs	0 (0.00)	0 (0.00)	0 (0.00)	0 (100)			
6–10 OFUs	0 (0.0)	6 (37.50)	10 (62.50)	16 (100)			
11–14 OFUs	15 (17.86)	33 (39.29)	36 (42.86)	84 (100)	0.13	−1.91	0.77
**GOHAI**							
GOHAI ≤ 45	7 (15.22)	19 (41.30)	20 (43.48)	46 (100)			
GOHAI > 45	8 (16.67)	17 (35.42)	23 (47.92)	48 (100)	0.85	0.20	0.96

Bold: statistically significant difference.

**Table 2:** Logistic regression model for the variables of periodontal disease and dental caries.

Variable	OR (SE) *	*p*-Value	95% CI
**Odds of having periodontal disease**			
married	26.25 (46.80)	0.07	0.797–864.56
urban location	0.03 (0.08)	**0.02**	0.00–0.36
non-smokers	0.05 (0.02)	0.07	0.00–1.55
high blood pressure	21.83 (37.83)	0.08	0.73–651.97
rheumatoid arthritis	25.33 (47.41)	0.08	0.65–992.63
diabetes	472.05 (158.57)	0.07	0.66–339,831.30
**Odds of having dental caries**			
men	0.31 (0.19)	**0.05**	0.09–1.01
urban location	3.02 (1.94)	0.09	0.86–10.66
diabetes	2.96 (2.43)	0.19	0.59–14.6
depression	3.05 (2.55)	0.18	0.60–15.65
never visited a dental hygienist	41.99 (0.05)	**<0.01**	0.01–0.38

* SE: Standard Error; Bold: statistically significant difference.

**Table 3:** Multinomial logistic regression model.

Variable	Relative Risk Ratio (se)	*p*-Value	95% Confidence Interval
**no caries, no periodontal disease**	base outcome		
**yes caries, no periodontal disease**			
age and gender	0.75 (0.30)	0.47	0.35–1.63
urban	2.17 (1.98)	0.39	0.37–12.90
drink alcohol regularly	2.45 (2.12)	0.30	0.45–13.33
has high blood pressure	2.72 (2.26)	0.23	0.53–13.89
has rheumatoid arthritis	12.80 (14.12)	**0.02**	1.47–111.20
use electric toothbrush	1.82 (1.46)	0.46	0.38–8.81
**no caries, yes periodontal disease**			
age and gender	0.97 (0.24)	0.91	0.60–1.58
urban location	0.33 (0.17)	**0.03**	0.12–0.92
drink alcohol regularly	1.68 (0.85)	0.30	0.62–4.52
has high blood pressure	2.53 (1.32)	0.08	0.91–7.03
has rheumatoid arthritis	6.91 (6.30)	**0.03**	1.16–84.00
use electric toothbrush	2.63 (1.32)	0.06	0.98–7.04

Bold: statistically significant difference.

## Data Availability

The data that support the findings of this study are available on request from the corresponding authors. The data are not publicly available due to privacy or ethical restrictions.
